# Effect of Framework Material and Thermal Aging on Shear Bond Strength of Three Different Gingiva-Colored Composite Resins

**DOI:** 10.3390/ma18235397

**Published:** 2025-11-30

**Authors:** Saliha Cagla Incearik, Guliz Aktas, Diler Deniz, Mustafa Baris Guncu, Mutlu Özcan

**Affiliations:** 1 Department of Prosthodontics, Faculty of Dentistry, Hacettepe University, Ankara 06100, Turkey; salihacagla@gmail.com (S.C.I.); cetin_diler@hotmail.com (D.D.); barisguncu@gmail.com (M.B.G.); 2 Center for Dental Medicine, Clinic for Masticatory Disorders and Dental Biomaterials, University of Zurich, 8032 Zurich, Switzerland; mutluozcan@hotmail.com

**Keywords:** adhesion, dental materials, gingiva-colored composites, titanium, zirconia, PEEK, shear bond strength

## Abstract

The purpose of this study was to evaluate the effect of different framework materials and thermal aging on the shear bond strength (SBS) of gingiva-colored composites used in fixed dental restorations. A total of 270 samples (10 × 10 × 2 mm^3^) were prepared using titanium, zirconia, and modified polyetheretherketone (modified PEEK). Three gingiva-colored composites (Gradia Gum, Anaxgum, Nexco) were applied after surface polishing and sandblasting. All specimens were stored in water at 37 °C for 24 h, then half of each group was subjected to thermal aging consisting of 10,000 cycles at temperatures between 5 and 55 °C. SBS testing was performed using a universal testing machine with a crosshead speed of 1 mm/min. Bonding failures were analyzed under a stereomicroscope, and one sample from each group was examined using a scanning electron microscope. SBS data were analyzed using three-way ANOVA with composite type, framework material, and thermal aging as factors, followed by pairwise comparisons (SPSS 23.0; *p* < 0.05). The highest SBS was recorded for the zirconia framework combined with Gradia Gum, specifically in the group without thermal aging (*p* < 0.05), while the lowest was observed for zirconia combined with Nexco after thermal aging (*p* < 0.05). Adhesive failures were predominant in the modified PEEK groups, whereas mixed failures occurred more frequently in titanium and zirconia groups. Both composite type and framework material significantly influenced SBS values, with thermal aging having a detrimental effect across all groups. This study demonstrates that both framework material and composite type affect bond strength, with specimens not subjected to thermal aging maintaining better adhesion. Thermal cycling reduced SBS in all groups, although the extent varied by material combination.

## 1. Introduction

Volume changes occur in soft and hard tissues following bone resorption after tooth extractions. Significant dimensional changes in hard and soft tissues may prevent optimal esthetics from being achieved solely through tooth restoration. White and pink esthetics should be evaluated together to achieve an optimal esthetic outcome [1]. Loss of soft tissues can be restored surgically [2]. However, surgical reconstruction may not always be the best option due to contraindications of the surgical procedure, possible disadvantages, or the patient’s unwillingness. In such cases, the replacement of the soft tissue with a gingiva-colored material integrated into the prosthetic restoration may be less invasive, relatively easier, and more acceptable for the patient [3].

In prosthetic restorations, the framework material must have sufficient physical and mechanical properties and must be compatible with the white and gingiva-colored veneer materials that will be applied on it [4]. A variety of materials, ranging from metal alloys to brand new materials, can be used as framework materials, with each offering its own advantages and disadvantages. Titanium and its alloys are widely preferred as framework materials [5], as titanium offers advantages such as low specific gravity, good corrosion resistance, high biocompatibility, mechanical properties similar to cast gold alloys, and low cost. Although titanium is very difficult to cast, its use frequency has increased with the rise in the milling method from CAD/CAM systems [6]. In addition, it has become possible to use zirconia—a non-cast ceramic material—as a framework material as a result of milling with CAD/CAM systems. Some of the advantages of zirconia include high biocompatibility, low bacterial adhesion, superior esthetics, and enhanced fracture resistance compared to metal [7]. Since certain types of zirconia are opaque, it must be veneered with a translucent material such as porcelain to achieve esthetic results [8]. In addition to titanium and zirconia, modified polyetheretherketone (modified PEEK) reinforced with ceramic additives has begun to be used as an alternative framework material produced with CAD/CAM in recent years [9]. Modified PEEK material is a polymer with high dimensional and thermal stability and good mechanical properties. However, due to its color and opacity, it cannot be used monolithically, especially with regard to esthetics, and a translucent veneering material is needed, similar to first- and second-generation zirconia [10].

The framework material should be covered with a gingiva-colored material to restore the gum prosthetically. For this purpose, gingiva-colored porcelains, acrylic resins, silicone-based materials, or composite resins can be used [11]. Gingiva-colored composites are often preferred because they offer several advantages, such as chair-side applicability; repairability; polymerization with light curing, which eliminates the need for firing; and predictable clinical results [12]. With an increasing frequency of preference for gingiva-colored composite resins, manufacturers have produced different gingiva-colored composite resin systems using different primers and adhesives. Since the gingiva-colored composite resin material that restores soft tissues in a prosthesis is not directly exposed to occlusal load, fewer complications can be expected. However, mechanical stress can be transmitted from the occlusal surface to the cervical region and cause fracture in the material [13]. Chipping of the veneering material in restorations is one of the problems frequently encountered in the clinic [14]. For the long-term success of a prosthetic restoration, there must be a strong bond strength between the framework material and the veneering material [15]. Evaluation and understanding of the physical properties of gingiva-colored composites are necessary for their correct use in clinical applications. Studies on the bonding strength of composite resins to other materials as well as on increasing this strength have been carried out for a long time. However, since most previous studies used tooth-colored composite resin, there are a limited number of studies on gingiva-colored composite resin in the literature [16,17]. The clinical importance of evaluating the adhesion of gingiva-colored composites to various framework materials arises from their direct impact on the durability and esthetic performance of implant-supported and removable prostheses. Achieving reliable bond strength is essential for preserving pink esthetics and minimizing the incidence of interfacial complications such as delamination, discoloration, and loss of structural integrity during function and aging [15]. Thus, the aim of this study was to evaluate the effect of different framework materials produced by CAD/CAM milling, currently used in fixed prosthetic restorations, as well as thermal aging on the SBS of gingiva-colored composite materials used to provide pink esthetics. The null hypothesis of this study was that there would be no differences in the SBS values based on the type of gingiva-colored composite, framework material, and thermal aging.

## 2. Materials and Methods

According to the power analysis, the sample size of the study was determined as *n* = 15, ensuring a significance level (α) of 0.05 (5%), a 95% confidence interval, and a test power (1 − β) of 0.9 (90%), calculated using G*Power software v3.1.9.7.

### 2.1. Preparation of Framework Specimens

Titanium, zirconia, and modified PEEK were used as framework materials (Table 1). Ninety samples with dimensions of 10 × 10 × 2 mm were prepared from each framework material. To ensure the standardization of the bonding surfaces, titanium and modified PEEK samples were polished under water with 600, 1000, and 1200 grit sandpapers, respectively. Zirconia samples were polished dry, and then the sintering process was carried out. To keep the samples stable, during the SBS test, the framework materials were fixed into auto-polymerizing acrylic resin (BMS Cold Acrylic, BMS Dental, Capannoli, Italy) using a silicone mold, leaving the bonding surfaces exposed. The bonding surface of the samples was sandblasted (Sefa San Dental Devices, İzmir, Türkiye) with 110 µm Al_2_O_3_ powders for 10 s at an angle of 45° from 10 mm, under a pressure of 3 bar for both titanium and zirconia and 1.5 bar for modified PEEK, in accordance with the manufacturer’s recommendations [18].

### 2.2. Application of Gingiva-Colored Resin Composite

#### 2.2.1. Application of Primer

The different framework samples were divided into three groups, and a gingiva-colored composite system (Gradia Gum, Anaxgum, Nexco) was applied to each group using the manufacturer’s recommended primer and gingiva-colored opaque (Table 1). After sandblasting, the bonding surfaces were cleaned with oil-free dry air, and the appropriate primer was applied: Gradia Gum group: G-Multi Primer was applied to both titanium and zirconia frameworks. Anaxgum group: Metal Bonder was applied to titanium frameworks, while Zircon Bonder was used for zirconia frameworks. Nexco group: SR Link was applied to both titanium and zirconia frameworks. For all gingiva-colored composite groups, Visio.link was applied as a very thin primer layer to the modified PEEK frameworks and polymerized for 3 min (an irradiance of approximately 2000 mW/cm^2^, a power output of 8–10 W, and an emission spectrum of 400–490 nm, Labolight LV-III, GC Europe, Leuven, Belgium) (Table 2).

#### 2.2.2. Application of Gingiva-Colored Opaque

To ensure precise application, a circular hole was punched into a masking tape using a rubber dam punch, limiting the application area. The masking tape with the hole was placed on the framework material. Two layers of gingiva-colored opaque material were then applied within this area. Each layer was polymerized separately (an irradiance of approximately 2000 mW/cm^2^, a power output of 8–10 W, and an emission spectrum of 400–490 nm, Labolight LV-III, GC Europe, Belgium). For the Nexco group, any excess oil on the polymerized layer was removed by gently pressing down with cotton gauze, and this process was repeated for both layers.

#### 2.2.3. Application of Gingiva-Colored Composite

The samples were placed in a specialized kit (Ultradent Bonding Clamp and Bonding Mold Inserts, South Jordan, UT, USA) designed for standardized bonding. This kit included a clamp to secure the sample and a mold with a diameter of 2.38 mm and height of 3 mm. The gingiva-colored composite was packed into the mold in two layers, with each layer being polymerized using a wireless light device with an irradiance of up to 2000 mW/cm^2^ and an emission spectrum covering 390–490 nm (GC D-Light Pro, GC Europe, Belgium). After removing the samples from the mold, a final polymerization process was conducted (an irradiance of approximately 2000 mW/cm^2^, a power output of 8–10 W, and an emission spectrum of 400–490 nm, Labolight LV-III, GC Europe, Belgium). Gingiva-colored composite application was performed by a single operator for all samples.

### 2.3. Thermal Aging and SBS Test

All specimens were first stored in distilled water at 37 °C for 24 h. Following this, half of the specimens (*n* = 15) were subjected to thermal aging with 10,000 thermal cycles (1 year of clinical aging) between 5 °C and 55 °C (±2 °C) (MOD Dental MTE-101, Esetron Mekanik, Ankara, Türkiye) [19], while the other half (*n* = 15) were tested without thermal aging.

**Figure 1:**
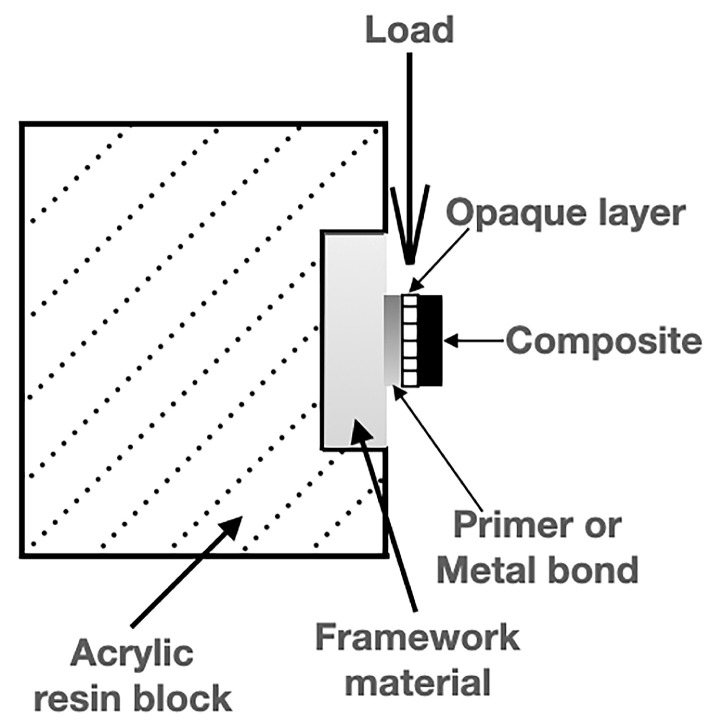
Schematic view of the test set up.

SBS testing was employed in the present study as it is one of the most standardized and widely used laboratory methods of assessing the initial interfacial adhesion between veneering resin composites and framework materials. This method provides reproducible and comparable data among different groups and has been commonly used in previous investigations of resin–framework bonding [20]. SBS testing was applied to all samples with a crosshead speed of 1 mm/min in a universal testing device (Lloyd LRX; Lloyd Instruments Ltd., Hants, UK). The shear load at the moment of failure in the connection area was recorded in Newton. The MPa value was calculated by dividing the shear load by the connection area of the bonding surface (Figure 1).

### 2.4. Analysis of Modes of Failure

Modes of failure were determined with a stereomicroscope (Olympus SZ61; Olympus Optical Co., Tokyo, Japan) at 40× magnification. The following modes of failure were noted: adhesive failure (AF); failure between opaque/framework interface, i.e., cohesive failure (CF); failure within composite resin and opaque only, i.e., mixed failure (MF); and failure between opaque/framework interface with failure within composite resin and opaque. One sample from each group was examined with an SEM device (GAIA3+Oxford XMax 150 EDS, Tescan Orsay Holding CZ, Brno, Czech Republic). For SEM analysis, sample surfaces were coated with 5 nm thick gold. SEM analysis image parameters were 6.00 kV, a light intensity of 7.00, an analysis scanning mode, and a magnification of 50×–200×.

### 2.5. Statistical Analysis

Since the data did not have a normal distribution, Box–Cox was used for all samples to maintain the normal distribution using the “AID” package in the R program v4.1.2 (R Core Team 2021) [21]. As the normality assumption was violated, a square root transformation (√x) was performed to approximate normal distribution prior to conducting the statistical analyses. SBS results were statistically analyzed via three-way ANOVA with the gingiva-colored composites, framework materials, and thermal aging as the independent variables. Pairwise comparison test was used to determine any significant differences among the groups. All statistical analyses were carried out with a special software (SPSS 23.0 for Windows; IBM Corp., SPSS Inc., Chicago, IL, USA) at a significance level of *p* < 0.05.

## 3. Results

A significant interaction was observed among the gingiva-colored composite resin, framework materials, and thermal aging, with thermal aging as the dominant influential factor. The second most influential factor was the gingiva-colored composite resin material, while the framework materials had the lowest impact overall (Table 3).

Pairwise comparisons as well as the mean and standard deviation values of all groups are presented in Table 3. In all groups, the thermal aging process resulted in a significant decrease in SBS values. Among the non-thermally aged samples, the highest mean SBS value was obtained from the group with a zirconia framework and Gradia Gum gingiva-colored composite. In contrast, the lowest mean SBS value was obtained from the group with a zirconia framework and Nexco gingiva-colored composite resin among the samples that were thermally aged. SBS values of the Nexco groups (as gingiva-colored composite), the modified PEEK groups (as framework material), and all thermally aged groups showed significantly lower SBS values than others (*p* < 0.05). The influence of varying gingiva-colored composites and framework materials on the SBS values is presented in Table 4.

**Figure 2:**
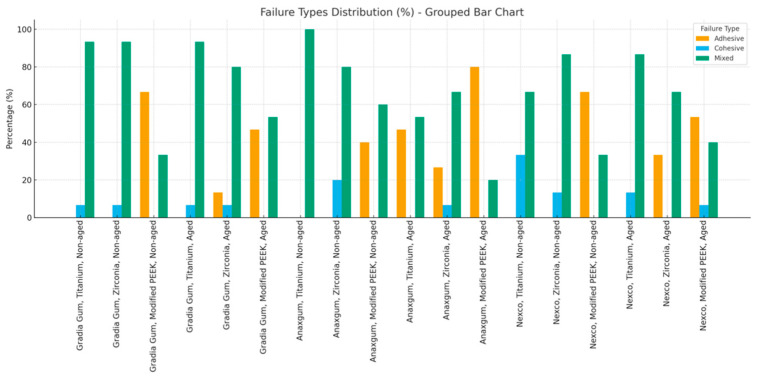
Distribution of failure types (adhesive, cohesive, and mixed) in non-aged and thermally aged groups for Gradia Gum, Anaxgum, and Nexco composites bonded to titanium, zirconia, and modified PEEK frameworks. Results are presented as percentages in grouped bar charts.

When the bonding surface of all samples was examined with a stereomicroscope after the SBS test, the adhesive, cohesive, and mixed failure rates were observed to be 26.3%, 6.7%, and 67%, respectively. While the adhesive failure rate was 19.25% for the non-thermally aged samples, this rate was 33.3% in the thermally aged samples. While the mixed type failure was predominant in the samples using titanium and zirconia frameworks (82.2% and 78.8%, respectively), adhesive type failure was more common in modified PEEK frameworks (58.8%). In the composite groups, Gradia Gum (74.4%), Anaxgum (63.3%), and Nexco (63.3%) showed mixed type failure at similar rates in total. In the Anaxgum gingiva-colored composite resin group, the adhesive failure rate was observed to be higher in the thermally aged group (46.6%) than in the non-thermally aged group (Figure 2, Table 5).

**Figure 3:**
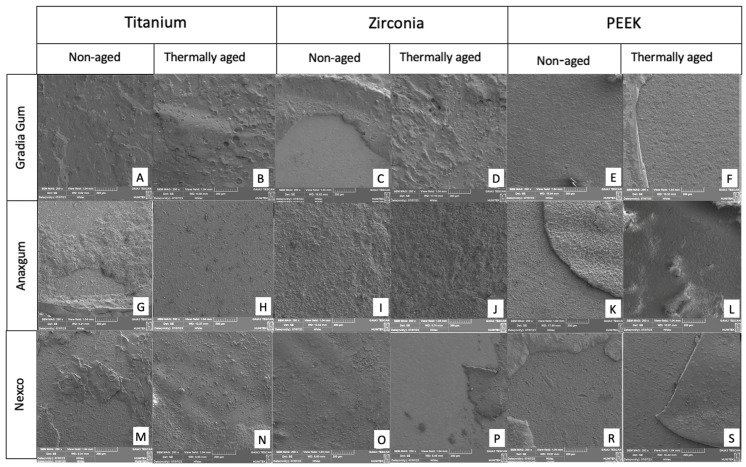
Representative SEM micrographs of fracture surfaces after shear bond strength (SBS) testing of gingiva-colored composite resins bonded to titanium, zirconia, and PEEK frameworks. Each horizontal row corresponds to one composite resin: Gradia Gum (**A**–**F**), Anaxgum (**G**–**L**), and Nexco (**M**–**S**). For each composite, specimens are shown for titanium (**A**,**B**,**G**,**H**,**M**,**N**), zirconia (**C**,**D**,**I**,**J**,**O**,**P**), and PEEK (**E**,**F**,**K**,**L**,**R**,**S**) frameworks under non-aged (**A**,**G**,**M**,**C**,**I**,**O**,**E**,**K**,**R**) and thermally aged (**B**,**H**,**N**,**D**,**J**,**P**,**F**,**L**,**S**) conditions. Different failure modes were observed: mixed (**B**,**C**,**G**,**K**,**M**,**P**,**R**,**S**), adhesive (**E**,**F**,**H**,**L**), and cohesive (**A**,**D**,**I**,**J**,**N**,**O**). Magnification: ×200; scale bar: 1 mm.

Representative SEM images of the fracture surfaces are presented in Figure 3. These micrographs illustrate the typical failure modes observed across different composite–framework combinations, providing a visual confirmation of the bond strength results.

## 4. Discussion

This study demonstrated that the framework material plays a crucial role in determining the SBS between gingiva-colored composite resins. Significant differences in SBS values were observed among titanium, zirconia, and modified PEEK frameworks, and thermal aging significantly decreased the bond strength in all groups. These findings led to the rejection of the null hypothesis.

The mean SBS value of the Gradia Gum composite with titanium and zirconia frameworks was similar, while modified PEEK showed lower SBS. The mean SBS value of Anaxgum composite with the titanium framework decreased much more than that of zirconia and modified PEEK frameworks. Moreover, the lowest SBS value was observed for the thermal aging group. The fact that the mean SBS values reduced more in the thermally aged Anaxgum composite with a titanium framework suggests that other agents which influence the bonding, such as the primer and opaque layer, are likely more sensitive to the thermal aging process. The mean SBS value of the Nexco composite was similar across all frameworks; however, a greater decrease in SBS values was observed with the zirconia framework in thermally aged groups. When titanium or zirconia was used as the framework material, the highest SBS values were obtained with the Gradia Gum composite. When modified PEEK was used as the framework material, similar SBS values were seen among all three composites, which could have resulted from using the same primer for this composite type. However, mean SBS values with modified PEEK frameworks were lower compared to other frameworks. The reason for this may have been an insufficient surface microporosity created by sandblasting the modified PEEK material at a pressure of 1.5 bar.

Thermal aging weakened the bond strength in all groups, in line with previous reports [22]. Beyond reducing initial bond values, aging introduces material-specific degradation pathways. Hydrolytic degradation can weaken phosphate- and silane-based bonds at the interface, while repetitive thermal aging accelerates crack propagation and interfacial fatigue [23,24]. These mechanisms highlight the multifactorial nature of adhesion failure.

Surface pretreatment is another critical factor. Different manufacturing methods of titanium, zirconia, and PEEK produce variable surface textures [25]. To minimize this effect, all samples in this study were pre-abraded with silicon carbide paper before sandblasting. Sandblasting with Al_2_O_3_ particles further increased surface roughness and exposed oxide sites for chemical interaction with primers [4,26]. However, the literature demonstrates wide variability in sandblasting parameters, with particle sizes and pressures ranging from 50 µm at 2 bar to more aggressive settings [27,28]. Such differences complicate cross-study comparisons and underscore the need for standardized protocols.

The findings of this study suggest that the adhesion of gingiva-colored composites may not be determined solely by the type of framework material but could also be influenced by the adhesive system and the intermediate opaque layer [29]. Among the adhesive components, MDP is of particular importance, as it chemically interacts with the oxide layers of zirconia and titanium, forming durable phosphate bonds that enhance the stability of the resin–framework interface [30,31,32,33]. In contrast, methacrylate-containing primers improve bonding to PEEK by modifying its surface characteristics [34]. Moreover, the application of an opaque layer may also affect the final bonding outcome, as its interaction with primers and adhesives can influence the chemical stability of the interface [34,35,36,37]. Therefore, the differences observed among the groups may be attributed not only to the intrinsic properties of the framework materials but also to the complex interplay of MDP-containing primers, methacrylate agents, and opaque layers.

Research by Lim et al. [38]. found that sandblasting titanium with 50 µm particles resulted in an SBS of 14.28 ± 2.62 MPa for the Ti-6Al-4V alloy and comparable results were observed for titanium frameworks with Gradia Gum and Anaxgum composite resins. An et al. [15]. examined the SBS of tooth- and gingiva-colored composite resins with feldspathic porcelain, base metal alloy, and zirconia. They found the highest SBS values for composite resin and feldspathic porcelain, while the lowest were with zirconia, regardless of composite color. Thermal aging did not significantly weaken SBS values for zirconia, possibly due to differences in sandblasting and primer agents [15]. Various surface treatments can be used in SBS evaluations. Çulhaoğlu et al. [29]. studied the effect of surface treatments on SBS between tooth-colored composite resin and modified PEEK material. Sandblasting at a pressure of 2 bar resulted in an SBS of 10.81 ± 3.06 MPa, whereas untreated samples had 5.09 ± 2.14 MPa. Thermal aging at 10,000 cycles yielded relatively low SBS values, likely due to lower-pressure sandblasting and the use of gingiva-colored opaque, which contains more resin monomers and fewer fillers. A 2022 study by Grover et al. investigated different surface treatments and found that sandblasting with a pressure of 5 bar resulted in an SBS of 4.58 ± 1.32 MPa [39]. Despite variations in sandblasting pressure and duration, SBS values for modified PEEK and gingiva-colored composites were similar to those in the present study.

When the bonding surfaces are examined with a stereomicroscope, more mixed type failures are observed with titanium and zirconia frameworks, while the most adhesive type failure was observed with modified PEEK frameworks, which may indicate that the gingiva-colored composite resin is less successful in bonding to the modified PEEK surface. These may have been affected by the lower bar pressure during the sandblasting process. When the gingiva-colored composite resins were compared among themselves in terms of failure types, they showed similar failure patterns. Considering the effect of thermal aging, an increase in the amount of adhesive failure was observed after thermal aging. This may indicate that the thermal aging is more effective in deteriorating the bond between the framework and the opaque material, and that this may represent a weaker link in the framework–opaque–composite resin bonding system [30,31,32,33].

The SEM observations supported the stereomicroscopic findings by revealing detailed surface characteristics of the fractured interfaces. Mixed failures were predominant in titanium and zirconia frameworks, where resin remnants and rough surfaces indicated strong chemical bonding through the MDP-containing primer, consistent with previous studies reporting durable phosphate–metal oxide interactions [30,31]. In contrast, smoother surfaces and higher adhesive failure rates in modified PEEK specimens suggested weaker interfacial adhesion due to its low surface energy and the lower sandblasting pressure applied during surface treatment, which may have limited surface activation [32,33]. When the gingiva-colored composite resins were compared, they showed similar failure patterns overall. After thermal aging, an increase in adhesive failures was observed, particularly in the Anaxgum group, indicating that thermal aging may weaken the bond between the framework and the opaque material, which represents the weakest link in the bonding system. These SEM findings were consistent with the shear bond strength results, confirming that both the framework material and primer composition play a decisive role in interfacial bonding performance.

Within the limitations of this study, only thermal aging was performed, which cannot fully reproduce intraoral conditions where restorations face combined hydrolytic, mechanical, and thermal stresses. Long-term clinical success also depends on factors such as hydrolytic stability of the resin matrix, resistance to fatigue loading, and color stability. Therefore, future investigations should incorporate combined thermal aging, fatigue tests, and extended storage, as well as explore alternative primers and adhesive–opaque combinations to optimize bonding strategies.

## 5. Conclusions

Within the limitations of this in vitro study, it can be concluded that the shear bond strength of gingiva-colored composite resins was influenced by the type of framework material. While titanium and zirconia frameworks showed variations depending on the composite resin used, modified PEEK exhibited similar values regardless of the composite type. Thermal aging significantly reduced bond strength in all groups, independent of the framework or composite. After thermal aging, adhesive failures predominated, especially in the PEEK groups. The highest bond strength values, both before and after thermal aging, were observed in certain composite–framework combinations involving titanium and zirconia. Clinically, careful consideration should be given when selecting PEEK as a framework material due to its limitations in bonding performance.

## Figures and Tables

**Table 1:** Used materials.

Product Used	Type of Product	Content	Manufacturer
Starbond Ti5 D isc	Grade 5 ‘Eli’ TiAl6V4 titanium alloy	Ti 89.4%, Al 6.2%, V 4%, N + C + H + Fe + O < 0.4%	Scheftner Dental Alloys, Mainz, Germany
Straumann ZI	Yttria-stabilized tetragonal zirconia (3 mol yttria content)	ZrO_2_ + HfO_2_ + Y_2_O_3_ ≥ 99.0%, Y_2_O_3_ 4.5–5.6%, ≤5% HfO_2_, Al_2_O_3_ ≤ 0.5%, other oxides ≤ 1%	Amann Girrbach, Koblach, Austria
breCAM.BioHPP^®^	Modified PEEK with ceramic additives	80%, PEEK 20% nanoceramic filler with particle size varying between 0.3 and 0.5 µm	Bredent GmbH, Senden, Germany
Gradia Gum Paste	Gingiva-colored composite	UDMA, dimethacrylate, inorganic fillers (71% by weight), prepolymerized fillers (6% by weight), photoinitiator, stabilizer, pigment	GC America, Inc. Alsip, IL, USA
G-Multi Primer	Metal and ceramic primer	Ethanol, MDP, γ-MPTS, MDTP, methacrylate monomer	GC, Tokyo, Japan
Gradia Gum Opaque	Gingiva-colored opaque material	Urethane dimethacrylate, silica, alumino-borosilicate glass, campharoquinone, pigment	GC, Tokyo, Japan
Anaxgum Gingiva Paste	Gingiva-colored composite	Urethane dimethacrylate, tetramethylene dimethacrylate, BisGMA, silicon dioxide, pigments, initiators, fillers (67% by weight 0.005–3.0 μm)	Anaxdent GmbH Stuttgart, Germany
Anaxdent Metal Bonder	Metal primer	Methyl methacrylate, phosphonic acid and macromers with sulfur groups	Anaxdent GmbH Stuttgart, Germany
Anaxdent Zircon Bonder	Ceramic primer	Methyl methacrylate, phosphonic acid, and macromers with sulfur groups	Anaxdent GmbH Stuttgart, Germany
Anaxgum Opaquer	Gingiva-colored opaque material	Di-urethane dimethacrylate, 2-butylaminocarbonyl oxyethyl acrylate, tetramethylene dimethacrylate, pigment initiators, silica powder	Anaxdent GmbH Stuttgart, Germany
SR Nexco Paste	Gingiva-colored composite	Dimethacrylate (17–19%), copolymer, stabilizers, catalysts, pigments (<1%), inorganic filler (43% by weight, 0.01–0.1 µm)	Ivoclar Vivadent, Schaan, Liechtenstein
SR Link	Metal and ceramic primer	Phosphoric acid group combined with methacrylate group, ethanol, benzol peroxide	Ivoclar Vivadent Inc. Amherst, NY, USA
SR NEXCO Gingiva Opaquer	Gingiva-colored opaque material	Dimethacrylate (65–70%), inorganic filler (<43%), catalyst, stabilizer and pigments (<2%)	Ivoclar Vivadent, Schaan, Liechtenstein
Visio.link	PEEK primer	MMA, dimethacrylate, pentaerythritol acrylate, photoinitiators	Bredent GmbH, Senden, Germany

**Table 2:** Application of primer.

		Zirconia	Titanium	Modified PEEK
Primary application	**G-Multi Primer**	A layer of primer was applied and allowed to interact for 15–20 s and dried with air.		x
	**Metal Bonder**	x	A layer of primer was applied and allowed to interact for 1 min and dried with air.	x
	**Zircon Bonder**	A layer of primer was applied and allowed to interact for 1 min and dried with air.	x	x
	**SR Link**	A layer of primer was applied and allowed to interact for 20 s and dried with air.		x
	**Visio.link**	x	x	Applied as a very thin layer, placed in a laboratory-type light device without drying, and polymerized for 3 min.

The ‘x’ indicates that this step is not performed for that material.

**Table 3:** Values of the three-way ANOVA test for shear bond strength results.

Source	Sum of Squares	df	Mean Square	F	*p*	Partial Eta Squared (η^2^)
Thermal Aging	186.382	1	186.382	289.286	<0.001	0.534
Gingiva-colored Composite	50.046	2	25.023	38.839	<0.001	0.236
Framework Material	20.786	2	10.393	16.131	<0.001	0.113
Gingiva-colored Composite * Thermal Aging	4.570	2	2.285	3.547	0.030	0.027
Framework Material * Thermal Aging	21.541	2	10.771	16.717	<0.001	0.117
Gingiva-colored Composite * Framework Material	30.879	4	7.720	11.982	<0.001	0.160
Gingiva-colored Composite * Framework Material * Thermal Aging	19.700	4	4.925	7.644	<0.001	0.108
Error	162.359	252	0.644			
Total	2963.646	270				

* indicates a relationship between the materials.

**Table 4:** Pairwise comparison, mean, and standard deviation values of the groups.

	Non-Aged Groups			Thermally Aged Groups		
	Titanium	Zirconia	Modified PEEK	Titanium	Zirconia	Modified PEEK
Gingiva-Colored Composite	Mean ± SD	Mean ± SD	Mean ± SD	Mean ± SD	Mean ± SD	Mean ± SD
Gradia Gum	16.35 ± 3.47	16.86 ± 3.56	8.17 ± 3.53	8.89 ± 3.01	8.42 ± 3.65	5.15 ± 1.90
	Aa	Aa	Ba	Aa *	Aa *	Ba *
Anaxgum	13.65 ± 4.36	12.41 ± 4.15	7.28 ± 1.94	2.89 ± 1.57	5.42 ± 3.24	4.60 ± 1.04
	Aa	Ab	Ba	Bb *	Ab *	Aa *
Nexco	8.81 ± 3.83	10.52 ± 3.14	8.29 ± 2.32	5.47 ± 1.40	2.70 ± 1.72	5.39 ± 1.46
	Ab	Ab	Aa	Ac *	Bc *	Aa *

SD: Standard deviation. Different capital letters in the horizontal direction indicate statistically significant differences in SBS values when the same gingiva-colored composite is applied on different frameworks in the same aging group (*p* < 0.05). Different lowercase letters in the vertical direction indicate statistically significant differences in SBS values when different gingiva-colored composites are applied on the same framework in the same aging group (*p* < 0.05). The presence or absence of an asterisk (*) in the horizontal direction indicates that there is a statistically significant difference in the SBS values of the groups before and after thermal aging when the same gingiva-colored composite is applied on the same framework (*p* < 0.05).

**Table 5:** Failure types.

		Non-Aged Groups			Thermally Aged Groups		
		Adhesive Failure	Cohesive Failure	Mixed Failure	Adhesive Failure	Cohesive Failure	Mixed Failure
Gradia Gum	Titanium	-	1	14	-	1	14
	Zirconia	-	1	14	2	1	12
	Modified PEEK	10	-	5	7	-	8
Anaxgum	Titanium	-	-	15	7	-	8
	Zirconia	-	3	12	4	1	10
	Modified PEEK	6	-	9	12	-	3
Nexco	Titanium	-	5	10	-	2	13
	Zirconia	-	2	13	5	-	10
	Modified PEEK	10	-	5	8	1	6

## Data Availability

The original contributions presented in this study are included in the article. Further inquiries can be directed to the corresponding author.

## Abbreviations

The following abbreviations are used in this manuscript:

SBS	Shear Bond Strength
CAD/CAM	Computer-Aided Design/Computer-Aided Manufacturing
PEEK	Polyetheretherketone
MPa	Megapascal
SEM	Scanning Electron Microscope
AF	Adhesive Failure
CF	Cohesive Failure
MF	Mixed Failure
ANOVA	Analysis of Variance
